# Cardiovascular Prevention and Rehabilitation for Ischaemic Non‐Obstructive Coronary Artery Disease: Implementation Considerations From a Survey of UK Health Professionals

**DOI:** 10.1111/jan.70023

**Published:** 2025-06-19

**Authors:** Simon Nichols, Susan Dawkes, Aynsley Cowie, Sarah Brown, Colin Berry, Helen Humphreys

**Affiliations:** ^1^ Centre for Cardiovascular Health Edinburgh Napier University Edinburgh UK; ^2^ School of Health and Social Care Edinburgh Napier University Edinburgh UK; ^3^ Cardiac Rehabilitation, NHS Ayrshire and Arran Kilmarnock UK; ^4^ The International Heart Spasms Alliance, Cardiovascular Care Partnership UK; ^5^ British Heart Foundation Glasgow Cardiovascular Research Centre, Institute of Cardiovascular and Medical Sciences University of Glasgow Glasgow UK; ^6^ Centre for Behavioural Science and Applied Psychology Sheffield Hallam University Sheffield UK

**Keywords:** ANOCA, cardiac rehabilitation, cardiovascular disease, cardiovascular rehabilitation, INOCA, microvascular angina, MINOCA, nursing, Prinzmetal angina, vasospastic angina

## Abstract

**Aims:**

Investigate if UK healthcare professionals have the resources and knowledge to provide cardiovascular prevention and rehabilitation to people with ischaemic non‐obstructive coronary artery disease (INOCA), and explore what type of care healthcare professionals believe patients should receive.

**Design:**

Electronic cross‐sectional survey of UK healthcare professionals, circulated between 7 January and 7 March 2022.

**Methods:**

Quantitative data were analysed descriptively. Qualitative data were analysed inductively.

**Results:**

Healthcare professionals lacked knowledge and capacity to care for this patient group. Healthcare professionals recommended patients receive two unsupervised sessions per week, for 8 weeks, at home and in person. Recommend include physical activity advice/exercise training, health behaviour support, psychological support, smoking cessation, dietetics/nutritional support, weight management, counselling and medication titration.

**Conclusion:**

In the UK, healthcare professionals lack resources and knowledge to provide cardiovascular presentation and rehabilitation to people with INOCA. Recommended care reflected care currently available to other patient groups.

**Implications for the Profession:**

There is a need to create and evaluate educational material for healthcare professionals.

**Impact:**

- Before people with INOCA are offered cardiovascular prevention and rehabilitation it was necessary to determine if healthcare professionals had sufficient clinical knowledge and resources to provide care.
- We conclude that additional training and resources are required to enable health professionals to deliver care to people with INOCA.

Researchers should create and evaluate educational material for cardiovascular prevention and rehabilitation programmes. Programmes also require additional resources to deliver care to this group.

**Reporting Method:**

Reporting adheres to the Cherries guidelines.

**Patient or Public Contribution:**

A patient (SB) was consulted on study design, data collection, and interpretation, and manuscript preparation.

**Summary**

- What does this paper contribute to the wider global clinical community?
  ○ We illustrate the support healthcare professionals need to provide cardiovascular prevention and rehabilitation to people with INOCA.
  ○ These data suggest how cardiovascular prevention and rehabilitation could be offered to this group.

## 1 Introduction

Cardiovascular prevention and rehabilitation (CPR), also known as cardiac rehabilitation, is a secondary prevention programme for people with heart disease and circulatory diseases (Cowie et al. 2019). In the UK, CPR typically includes nutritional support/weight management, psychosocial health management, smoking cessation, medical management and exercise training (Cowie et al. 2019). In coronary heart disease (CHD), CPR participation reduces cardiovascular deaths over 3 years (Dibben et al. 2021) and hospital admissions over 2 years (Powell et al. 2018). Similarly, CPR reduces hospital admissions in people with heart failure (HF) (Taylor et al. 2019) and improves health‐related quality of life (HRQoL), over 12 months (Hurdus et al. 2020). As well as providing CPR to traditional cohorts, such as CHD and HF, new guidance recommends CPR for people with other heart conditions, such as ischaemic non‐obstructive coronary artery disease (INOCA) (British Association for Cardiovascular Prevention and Rehabilitation 2023).

## 2 Background

Ischaemic non‐obstructive coronary artery disease is an umbrella term for endothelial dysfunction, microvascular and epicardial spasm and vasomotor abnormalities (Rahman et al. 2019). Approximately 46% of people who have an angiogram may have INOCA, rather than CHD (Ford et al. 2018). Having INOCA increases the risk of developing HF with preserved ejection fraction (Taqueti et al. 2018), and myocardial infarction at 1‐year (Maddox et al. 2014). Further, people with INOCA are 10% more likely to have depression than people with CHD (Wheeler et al. 2013). The risk factors and causes of INOCA are complex but risk factors, such as hypertension smoking, and diabetes predict major cardiovascular events and all‐cause mortality (Nordenskjöld et al. 2018). A comprehensive CPR programme, contemporary medical management (Kunadian et al. 2020), could address these risk factors. Indeed, small scale data indicate that exercise training improves HRQoL, depression symptoms, and delays the onset of angina symptoms in people with INOCA (Kissel and Nikoletou 2018). Exercise training and weight management may also reduce body mass, triglycerides, total cholesterol and HbA1c (Bove et al. 2020).

## 3 The Study

With CPR guidelines now suggesting that CPR could benefit people with INOCA (British Association for Cardiovascular Prevention and Rehabilitation 2023) there is a need to determine if CPR programmes have the capacity and capability to provide care.

### 3.1 Aim

This survey of UK healthcare professionals (HCPs) aimed to explore whether existing UK CPR programmes have the resources and knowledge to provide care to patients with INOCA.

### 3.2 Secondary Objectives

We also sought to establish what care HCPs believe should be available within a CPR programme for people with INOCA, and the content of a future clinical trial of CPR for people with INOCA.

## 4 Methods

### 4.1 Design

The methods and results are reported in conjunction with the EQUATOR Network Checklist for Reporting Results of Internet E‐Surveys (Cherries) (Kunadian et al. 2020) (Appendix S1). This was a voluntary, cross‐sectional, open survey, developed by the study team. The questionnaire focused on:

- Whether CPR programmes have the resources to include people with INOCA.
- Whether HCPs have the knowledge to treat people with INOCA.
- What care HCPs think should be provided to people with INOCA.
- Whether CPR programmes would participate in a future definitive multi‐centre RCT of CPR for people with INOCA.

The survey was developed by SN and was tested for relevance, functionality, clarity and accuracy by all members of the study team. This included a patient expert. Revisions to the survey were made in response to feedback. The survey was subsequently independently reviewed and piloted by eight members of the British Association for Cardiovascular Prevention and Rehabilitation (BACPR). Reviewers were instructed to assess for relevance, functionality and clarity, but no specific review criteria were used. The BACPR council consists of people in a variety of HCP roles which reflect the intended survey respondents. Feedback from this independent review led to further revisions of the questions and survey formatting. As the purpose of this survey was to gather HCP insights, further psychometric validation was not conducted. The resulting 37‐item (excluding consent questions) questionnaire was uploaded to the Qualtrics XM online survey platform (Provo, Utah, USA). Qualtrics has ISO/IEC 27001 security certification. The automated database was password‐protected and stored on secure Qualtrics and Sheffield Hallam University servers. No personal identifiable information was collected.

The survey (Appendix S2) was presented across eight pages, including background information and consent. There were 1–6 questions per page with 26 tick box items (25 mandatory) with the option ‘other’ for free text responses to eight of the questions. Additionally, there were four mandatory numerical responses, one mandatory sliding bar response and six non‐mandatory free‐text responses. Free text responses were included to obtain deeper insights into the perspectives of respondents. Response validation was used on all questions, where appropriate. Survey progress was displayed on each page. Participants did not have a completeness check/review option at the end of the survey but could move backwards and forwards manually to review/amend their answers. Questions were not randomised but adaptive questioning was used. Participants were only able to complete the survey once when accessing it using the same internet protocol (IP) address and had 14 days to complete the survey from that IP address once started. Although this could exclude some respondents attempting to complete the survey from a shared PC terminal, this was necessary to minimise duplicate responses and maintain data quality. The specific location of each respondent's CPR programme was not requested. To retain as much information as possible, partially completed surveys were included for analysis.

### 4.2 Study Setting and Sampling

This online survey targeted a convenience sample of HCPs working in a Core UK CPR programme (also known as Phase III cardiac rehabilitation).

### 4.3 Inclusion Criteria

To participate in this survey, respondents needed to be a UK HCP working in a Core CPR (Phase III) programme. Participants who had previously completed the survey were excluded. There were no other inclusion or exclusion criteria. These criteria were established at the start of the survey. Respondents who reported not fulfilling these requirements were prevented from completing the survey.

### 4.4 Data Collection

On 7 January 2022, a recruitment email was sent to BACPR members; 931 HCPs and academics working in CPR. This was repeated on 14 January and 25 February 2022 (Appendix S3). The survey was also promoted on social media platforms (Appendix S3). A survey link was not posted on any website. The survey closed at 23:59 on 7 March 2022. There were no incentives offered for participation.

### 4.5 Data Analysis

Data were exported into SPSS v24 (IBM, New York, NY, USA). The number of responses to each question varied due to attrition/non‐compulsory questions. Thus, responses (%) are expressed as a percentage of the number of respondents to each question. The total number of respondents to each question are noted in the narrative and is also reported in each table/figure. Continuous data are reported as median, with minimum and maximum values, or as mean and standard deviation (±) when specified. Spearman correlation was used to explore the relationship between continuous variables. Otherwise, inferential statistics were not used because this study was exploratory. Further, the study employed convenience sampling, which does not guarantee a representative sample of the population. Additionally, no hypothesis testing or sample size calculation was performed, as the primary aim was to generate insights rather than statistical generalisability. Complete and incomplete surveys were categorised into binary variables and entered into backwards stepwise binary logistic regression to explore whether data were missing at random. Respondent characteristics, and the total time spent completing the survey, were used as independent variables.

For qualitative data, where respondents had provided optional free‐text answers, these were exported into NVivo V.14 for thematic analysis. Answers were coded inductively by a single researcher. In keeping with the descriptive nature of the study, the researcher undertook ‘semantic’ style open coding, as described by Braun and Clarke (2013) aiming for codes to provide a descriptive summary of the response rather than deeper interpretation of meaning. Similar codes were grouped to form lower order and subsequently higher order themes summarising the key points. Coding was undertaken by a single researcher (SN), who kept a reflexive journal throughout theme development. Preliminary themes were discussed with two other researchers experienced in qualitative analysis to enhance researcher reflexivity and trustworthiness. Each theme was given a description, and illustrative quotes were provided.

The authors affirm that the methods used in the data analyses are suitably applied to their data within their study design and context, and the statistical findings have been implemented and interpreted correctly.

### 4.6 Ethical Considerations

This work was funded by the British Heart Foundation Clinical Research Collaborative. The funder took no part in the design or conduct of the study. Sheffield Hallam University provided institutional ethical approval (ID: ER37686828) on 5th November 2021. All participants provided informed consent.

## 5 Results

### 5.1 Characteristics of the Sample

There were 189 visits to the survey site. Consent was provided by 155 (82.0%) respondents. However, 31 (20.0%) did not work in Core CPR, five (3.3%) had previously completed the questionnaire, and five (3.3%) did not work in the UK. Thus, 41 (26.5%) respondents were excluded and 114 were eligible to progress to the survey questions. Six respondents chose not to progress leaving 108 survey responses for analysis.

Participants took 11.7 min (±8.4 min) to complete the survey. Of the 108 respondents, *n* = 85 (78.7%) completed the survey in full. Backward stepwise binary logistic regression, used to explore whether survey data were missing at random, eliminated all predictor variables from the model, leaving a constant (*B* = −1.699, *p* < 0.001). This suggests that survey completion/non‐completion was not associated with any of the predictors in the model. A pragmatic decision was made to undertake complete case analysis. Of 108 responses, *n* = 74 (68.5%) were from England. Fewer responses were received from Scotland (*n* = 18; 16.7%), Wales (*n* = 11; 10.2%) and Northern Ireland (*n* = 5; 4.6%). Respondents had been working in CPR for 12.8 ± 3.8 years and were most commonly nurses (*n* = 56; 51.9%), physiotherapists (*n* = 27; 25.0%) or exercise physiologists (*n* = 11; 10.2%) (Appendix S4).

### 5.2 Cardiovascular Prevention and Rehabilitation Programme Characteristics

A total of 102 responses to questions related to the components of CPR that were currently available were received. Typically, CPR programmes offered physical activity advice (*n* = 101; 99.0%), exercise training (*n* = 100; 98.0%), health behaviour change support (*n* = 95; 93.1%), psychological support (*n* = 90; 88.2%), dietetics or nutritional support (*n* = 77; 75.5%) and smoking cessation (*n* = 76; 74.5%). Medication titration (*n* = 68; 66.7%), weight management (*n* = 56; 54.9%), counselling (*n* = 51; 50.0%), relaxation (*n* = 1; 1.0%) and occupational therapy (*n* = 1; 1.0%) were offered less frequently.

**FIGURE 1:**
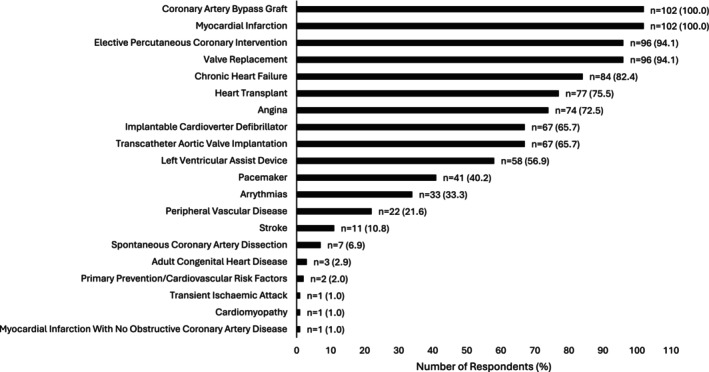
Conditions that respondents' cardiovascular prevention and rehabilitation programmes currently accept referrals for. Bars represent number of respondents (% of respondents; total question responses = 102).

The cardiovascular conditions CPR programmes stated they would accept referrals for are shown in Figure 1. One respondent accepted referrals for people with a primary diagnosis of myocardial infarction with no obstructive coronary artery disease (1.0% [note: this is different to INOCA]). When directly asked if patients with a primary diagnosis of INOCA were accepted by a CPR programme, 26 (25.5%) said no. Remaining respondents rarely accepted INOCA referrals (*n* = 41; 40.2%), accepted several referrals each year (*n* = 26; 25.5%) or each month (*n* = 7; 6.9%). Two (2.0%) accepted referrals at least weekly. Sixty‐seven respondents stated how INOCA referrals were accepted. Most INOCA referrals were ad‐hoc (*n* = 44; 65.7%) rather than through a formal referral pathway (*n* = 23; 34.3%).

### 5.3 Resources

Most respondents believed people with INOCA would benefit from participating in CPR (*n* = 77; 88.5%; total question responses *n* = 87). Though 11.5% were unsure, no respondents stated CPR would not be beneficial. Centre‐based or home‐based programmes were both considered appropriate CPR delivery formats (*n* = 66; 72.5%; total question responses *n* = 91). However, few respondents believed their service had capacity to assess (*n* = 25; 27.5%) or treat (*n* = 23; 25.3%) people with INOCA (Table 1). Lack of staff/human resources (*n* = 66; 69.5%), funding (*n* = 58; 61.1%) and space/CPR venues (*n* = 44; 46.3%) were cited as factors (total question responses *n* = 95). Less commonly, additional equipment was thought to be required (*n* = 17; 17.9%). HCPs also wanted better INOCA‐specific cardiovascular risk factor education (*n* = 1; 1.1%), training on INOCA (*n* = 1; 1.1%), and closer support from a cardiologist (*n* = 1; 1.1%). Two respondents did not say what resources were required (*n* = 2; 2.1%), and 13 (13.7%) believed no additional resources were required.

**TABLE 1:** Responses to questions about whether cardiovascular prevention and rehabilitation programmes have the capacity to provide services to patients with ischaemic non‐obstructive coronary artery disease.

(Q) If there was a formal referral process for patients with ischaemic non‐obstructive coronary artery disease would you have the capacity to assess them? (*n* = 91 total responses)	Responses (%)
Yes—There would be no problems with capacity	25 (27.5)
Yes—But we would only have capacity for a limited number of patients	28 (30.8)
No—We would not have capacity to see them	21 (23.1)
Not sure	17 (18.7)

(Q) If there was a formal referral process for patients with ischaemic non‐obstructive coronary artery disease, would you have the capacity to provide comprehensive cardiovascular prevention and rehabilitation to them? (*n* = 91 total responses)	Responses (%)
Yes—There would be no problems with capacity	23 (25.3)
Yes—But we would only have capacity for a limited number of patients	35 (38.5)
No—We would not have capacity to see them	21 (23.1)
Not sure	12 (13.2)

(Q) How would the cardiovascular prevention and rehabilitation programme need to be delivered? (*n* = 91 total responses)	Responses (%)
Centre or home‐based (including technology)	66 (72.5)
Centre‐based only	2 (2.2)
Home‐based only (including technology)	3 (3.3)
Not sure	20 (22.0)

*Note:* Number of responses reported, with percentages.

### 5.4 Knowledge

**FIGURE 2:**
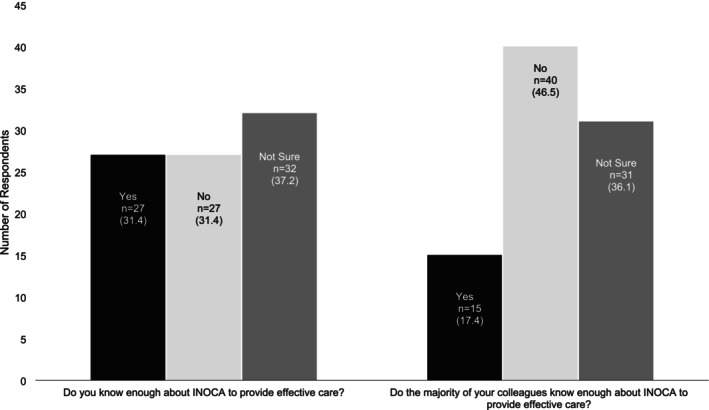
The number (%) of respondents who believe they (left) or their colleagues (right), know enough about ischaemic non‐obstructive coronary artery disease to provide an effective CPR programme (total question responses = 86).

Only 27 (31.4%) respondents believed they had enough knowledge about INOCA to provide effective CPR (total respondents *n* = 86). Even fewer (*n* = 15; 17.4%) believed the majority of their colleagues did (Figure 2). Despite this, 66 (77.0%) said they had previously provided CPR to someone with INOCA. Of these, only 14 respondents (21.2%) said they were completely confident in doing so, 47 (71.2%) would have liked additional training, and five (7.6%) explicitly stated they were not always able to provide a good level of care. When rating their confidence about managing patients with INOCA on an arbitrary scale of 0–10 (10 being completely confident), the median response was 7 (range 3–10). Both responses from doctors (100%), 44% (*n* = 4) from exercise physiologists, 31.9% (*n* = 15) from nurses, 25.0% (*n* = 5) from physiotherapists and 33.3% (*n* = 1) from ‘other’ respondents indicated they had sufficient knowledge to care for people with INOCA. The remaining professions, including a dietician (*n* = 1), an occupational therapist (*n* = 1) exercise instructors (*n* = 3) said they did not. There was no significant correlation between the total number of years working in CPR, and confidence managing patients with INOCA (*r* = 0.192; *p* = 0.122).

If required, respondents said they would improve their knowledge about INOCA by attending a continuing professional development (CPD) course (*n* = 78; 83.9%), reading healthcare Journals (*n* = 71; 76.3%), speaking to colleagues (*n* = 62; 66.7%), consulting the internet (*n* = 54; 58.1%), or by signing up for a university module (*n* = 33; 35.5%; total question responses *n* = 93). One (1.1%) said they would consult existing CPR guidelines, and another (1.1%) said that they already had enough knowledge. One respondent (1.1%) did not consider this question to be applicable.

### 5.5 Future Trial Design

**FIGURE 3:**
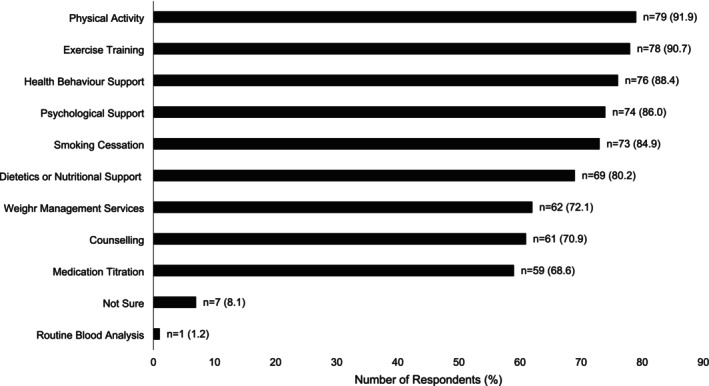
The number (proportion %) of respondents stating that an individual component of cardiovascular prevention and rehabilitation should be offered to patients with ischaemic non‐obstructive coronary artery disease in a research trial (total question responses = 86).

Forty‐nine HCPs (57.6%) stated that their CPR programme would be willing to participate as a research centre in a future funded clinical trial (total question responses *n* = 85). Thirty HCPs were unsure (35.3%) and six were unwilling (7.1%). When asked about the design of a research trial of CPR for people with INOCA, respondents believed that CPR should be delivered over a median of 8 weeks (range 0–26 weeks). Most believed that a hybrid of centre‐ and home‐based CPR would be most appropriate (*n* = 70; 80.5%; total question responses *n* = 87). Four said CPR should be home‐based (4.6%), one favoured centre‐based (1.1%), and 12 were unsure (13.8%). The CPR components that respondents believed should be offered to people with INOCA are shown in Figure 3. Respondents were least likely to consider medication titration (*n* = 59; 68.6%), counselling (*n* = 61; 70.9%), weight management services (*n* = 62; 72.1%), or routine blood analysis within the scope of a research trial (*n* = 1; 1.2%).

Thirty‐three respondents (37.9%; total question responses *n* = 87) were unsure how many weekly supervised CPR sessions people with INOCA should participate in. From the remaining responses, two supervised CPR sessions per week were recommended (range 0 to 16 sessions). After excluding 22 implausible responses (8–16 sessions), two supervised CPR sessions per week were still recommended (range 0–6 sessions). Respondents recommended three weekly *unsupervised* CPR sessions (range 3–24). After excluding six implausible responses (range 8–24 sessions per week), two unsupervised weekly CPR sessions were recommended (range 0–5 sessions). Forty‐four respondents (50.6%) were unsure how many unsupervised CPR sessions people with INOCA should participate in.

### 5.6 Qualitative Results

Qualitative analyses were undertaken for each free text response within the survey. Higher order themes (Appendix S5) are discussed below.

### 5.7 Resources

When asked, some respondents reported having sufficient resources to assess patients with INOCA, whilst others did not. People with sufficient resources cited reasons including their service expanding, having enough staff, or anticipating a small number of patients with INOCA being referred.

I would assume numbers would be low, and we could therefor absorb them into our caseload

In contrast, respondents who did not have sufficient resources cited insufficient staff or clinical space, already having long waiting lists, lack of funding, or that COVID‐19 measures were still limiting service capacity.

We don't offer comprehensive rehab to our current cohort so would need more staff e.g., psychology, dietetics, and access to space for clinics and exercise

A lack of established referral process, or ad‐hoc referral processes, was cited as a barrier to having sufficient assessment capacity. Some respondents were unclear how many patients would need to be assessed so were unsure whether they would have sufficient resources.

There is no local data on the number of patients with INOCA who would be eligible for CR

Similar to assessment, insufficient resources, including too few staff, limited clinical space, long waiting lists and insufficient funding all limited capacity to provide CPR to people with INOCA. An expanding CPR workforce or envisaging a low number of INOCA referrals were, again, factors contributing to programmes having sufficient resources to provide CPR. Existing referral processes was also a reason cited by some for having capacity to treat people with INOCA. Logistical factors including unclear patient demand, or health service commissioning arrangements being unclear, were cited as reasons for services being unsure if they would have capacity. Uncertainty about how to personalise patient‐facing educational material was cited as a barrier to having capacity to treat patients with INOCA.

Better tailored education as conventional CV risk management will not be relevant to all in this group

### 5.8 Programme Format

Respondents commonly reported that centre‐based, home‐based, or hybrid CPR should be made available to patients, based on their preference, ability, or clinical risk.

They should have the choice of all options, but anecdotal experience suggests our patients benefit most from a combination of group and home based

Some responses were also based on what CPR programmes were already offering. In these instances, responses were binary: face‐to‐face CPR or home‐based. Lack of resources, including clinical space, caused some respondents to say that CPR should be exclusively home‐based.

Home‐based is only available at the moment

## 6 Discussion

The aim of this survey was to investigate whether UK HCPs have the resources and knowledge to provide CPR to people with INOCA. We also sought their views about what level of care should be available to people with INOCA who participate in CPR routinely, or as part of a future clinical trial. We received 108 eligible responses from all four UK nations. Respondents believed that people with INOCA should participate in CPR twice per week for 8 weeks, reflecting the typical frequency and duration of CPR in the UK (National Audit of Cardiac Rehabilitation 2019). Respondents also believed that CPR should entail physical activity advice, exercise training, behaviour change support, psychological support and smoking cessation. Dietetics or nutritional support, weight management, counselling and medication titration were recommended less frequently. Although face‐to‐face and home‐based CPR were both considered appropriate, most respondents stated that they would be unable to provide CPR for those with INOCA due to a lack of resources. Furthermore, few respondents had sufficient knowledge about INOCA to deliver CPR. Thus, if CPR were to be provided to patients with INOCA, additional funding and training would be required.

### 6.1 Service Capacity to Treat People With INOCA

Some respondents reported having formal CPR referral processes for people with INOCA. This may partially explain why some respondents thought they would be able to assess and treat patients with INOCA, whilst others did not. However, qualitative data highlighted uncertainty about how many patients would need CPR. This created further uncertainty about whether CPR programmes would have sufficient resources to assess/treat patients with INOCA. The number of people requiring CPR is an important consideration for future health service and clinical trial design.

With the publication of guidance by the European Association of Percutaneous Cardiovascular Interventions (Kunadian et al. 2020), and evidence suggesting that approximately 46% of patients undergoing angiogram have INOCA (Ford et al. 2018), the number of INOCA diagnoses is expected to rise substantially. This anticipated increase highlights the urgent need to develop global CPR infrastructure for INOCA, which will likely require significant investment. In the USA, over one million angiograms are performed annually, suggesting that ~460,000 people with INOCA could require CPR. In the UK, more than 155,000 people undergo an angiogram each year (Asher et al. 2019), suggesting approximately 71,300 additional UK patients with INOCA could become eligible to participate, each year. Of those, based on existing participation rates (National Audit of Cardiac Rehabilitation 2019), half will enrol (~35,650). Additional demand may arise where individuals with INOCA experiencing symptom exacerbation and requiring further input from CPR teams. Therefore, the demand for CPR among people with INOCA may surpass that seen in other cardiac populations. However, the diagnosis of INOCA is often delayed and uncoordinated. Patients can live with symptoms for up to 3 years before being diagnosed (Gulati et al. 2023), experience high levels of depression (Wheeler et al. 2013), reduced HRQoL (Gulati et al. 2023) and often struggle to access suitable care (Humphreys et al. 2024). Whilst some individuals may see CPR as a valuable opportunity to access healthcare, others may have disengaged with healthcare services. Patient willingness to participate in CPR is therefore unclear.

### 6.2 Level of Care

In CHD, face‐to‐face and home‐based CPR are both effective at reducing the risk of myocardial infarction and improving HRQoL (Dibben et al. 2021; Powell et al. 2018). In our survey, respondents reported that face‐to‐face or home‐based CPR would also be appropriate CPR delivery modes for people with INOCA (72.5%). People with INOCA often have unpredictable and recurrent symptom exacerbation. Providing a choice of how patients access CPR may increase patient participation. Increasing participation in, and completion of, CPR has been an international priority for many years (Clark et al. 2004; Beatty et al. 2023) and became healthcare policy in England in 2019 (NHS England 2019). Opportunities to maximise participation in CPR should be considered before its universal implementation. Furthermore, allowing patients to freely switch between face‐to‐face and home‐based CPR could help them access support, even when they are experiencing symptom exacerbation (Humphreys et al. 2024). This could improve patient HRQoL and reduce the risk of hospitalisation.

In CHD and HF, the benefits of CPR are widely accepted to be achieved through a comprehensive programme of education and risk factor management (Ambrosetti et al. 2021). In our survey, HCPs recommended many traditional CPR components for people with INOCA (Figure 3). However, ranked recommended components of CPR did not always follow evidence. For example, fewer respondents thought that weight management should be included (72.1%). Weight management, in conjunction with exercise training, may reduce angina frequency/severity in people with coronary microvascular dysfunction (Bove et al. 2020). Further, despite the importance of medical management (Ang and Berry 2021; Kunadian et al. 2020), medication titration was recommended less frequently for people with INOCA (68.6%). Similarly, patients with INOCA have higher levels of depression than those with CHD (Wheeler et al. 2013). Yet, 29.1% of HCPs did not recommend counselling for people with INOCA. It is possible that some respondents thought ‘psychological support’ and ‘counselling’ were similar and only selected one of the options. However, the components that HCPs recommended may have been based on what they were already able to offer their patients, rather than what might be beneficial. Our quantitative data support this as the CPR components that HCPs recommended for people with INOCA were similar to the components they were already offering to other patients. Therefore, our findings could indicate a lack of weight management, medication titration and counselling services in existing UK CPR programmes. Respondents self‐reported lack of knowledge about what could benefit people with INOCA could also be a factor.

### 6.3 Education and Training

Over two thirds of respondents (71.2%) said they would like additional training to help them care for people with INOCA. However, there were conflicting data on whether HCPs were confident providing care to patients with INOCA. Uncertainty about how to personalise CPR material for patients was cited as a barrier to having capacity to treat patients with INOCA, and few respondents believed they (31.4%) or their colleagues (17.4%) had appropriate knowledge to provide CPR to people with INOCA. However, when asked to arbitrarily rate their confidence on a scale of 0 to 10, respondents median score was seven, indicating greater confidence (10 = completely confident). The lack of knowledge appeared independent of CPR experience and, with the notable exception of doctors, was common across all professions. A lack of knowledge about INOCA may explain why some CPR components that may benefit people with INCOA were not always recommended. Indeed, previous work identified that HCPs working in Europe also lacked sufficient knowledge to treat people with INOCA (Van Schalkwijk et al. 2023). This led to patients with INOCA being treated in a similar way to patients with other types of heart disease, patients not completing CPR or suffering burnout, and some cardiologists advising patients not to participate in CPR (Van Schalkwijk et al. 2023). Thus, workforce training appears essential before CPR is offered to people with INOCA. Our respondents favoured participating in a CPD course (83.9%). Previous randomised controlled trials of CPR for heart failure, such as REACH‐HF (Dalal et al. 2019), have successfully provided short training courses for UK HCPs (Dalal et al. 2019). Similar courses could be co‐developed for HCPs wanting to deliver CPR for people with INOCA training to HCPs working in CPR.

### 6.4 Limitations

Our open‐ended questions provide qualitative insights into the opinions of UK HCPs. Whilst we took steps to ensure our data were reported transparently, a single researcher conducting inductive analysis could lead to interpretation bias. We took steps to minimise this bias by engaging in reflexive and transparent practices but acknowledge that further researcher triangulation or multiple coders could have enhanced this process further. Future research may wish to use our findings to enable appropriate use of inferential statistics and enable the ‘significance’ of differences and associations to be reported.

We were also unable to report how many responses came from individual CPR programmes. Therefore, it is possible that a disproportionately high number of responses were received from a small number of CPR programmes. Further, we used convenience sampling to recruit participants to this study. The opinions of the participants electing to participate may therefore not be fully representative of UK CPR programmes. We also included data from partially completed surveys to maximise all available data. Factors such as the length of the survey could have contributed to incomplete surveys. However, the average time taken to complete the survey (11.7 min) was within the 15‐min survey duration that we described in our participant information sheet. Additionally, our data exploration suggests that responses were likely missing at random, but there remains a possibility of a response bias. Despite these limitations, HCPs may rarely encounter this patient group, making it reasonable to suggest they lack the knowledge and resources to provide optimal care. Furthermore, with limited research on CPR for people with INOCA, HCPs may rely on personal case studies, assumptions that INOCA patients have similar needs to other groups, or the belief that all patients benefit from holistic lifestyle interventions. Thus, our findings likely reflect the current state of UK CPR. There is a clear need to explore perspectives from expert clinicians and patients with lived experience to define an effective CPR programme for people with INOCA.

While our study did not set out to examine whether factors such as staff seniority, programme region, or other programme‐level factors influence the capacity or knowledge required to deliver CPR to people with INOCA, we recognise this as a potentially important area of future investigation. This will be particularly important if certified training on INOCA becomes available for CPR professionals, or people with INOCA become routinely eligible for CPR.

## 7 Conclusion

In the UK, HCPs working in CPR programmes are willing to provide care to people with INOCA in routine practice and/or in a clinical trial. However, funding, other physical resources and referral infrastructure are required. There is evidence that HCPs are willing to be led by the needs of the patient and use face‐to‐face or home‐based delivery modes, as needed. This will bevital for improving uptake and participation. We found that HCPs believed many traditional components of CPR should be offered to patients with INOCA, but further work is needed to determine which of those components are underpinned by research and patient demand. Whilst our data can only be interpreted within the context of UK healthcare systems, our data provide sufficient rationale for researchers working in other countries to establish whether their workforce is equipped to provide adequate care to people with INOCA. In the UK, however, well‐designed training is needed to enable HCPs to deliver high‐quality CPR to people with INOCA. These factors should be considered before CPR is delivered to people with INOCA in routine clinical practice or in a clinical trial.

## Ethics Statement

Sheffield Hallam University provided institutional ethical approval (ID: ER37686828) on 5th November 2021.

## Consent

All participants provided informed consent.

## Conflicts of Interest

The authors report details of affiliation or involvement in an organisation or entity with a financial or non‐financial interest in the subject matter, or materials discussed in this manuscript, in the attached ICMJE conflicts of interest disclosure form.

## Supporting information


Appendix S1.

Appendix S2.

## Data Availability

The data that support the findings of this study are available from the corresponding author upon reasonable request.

## References

[jan70023-bib-0001] Ambrosetti, M. , A. Abreu , U. Corrà , et al. 2021. “Secondary Prevention Through Comprehensive Cardiovascular Rehabilitation: From Knowledge to Implementation. 2020 Update. A Position Paper From the Secondary Prevention and Rehabilitation Section of the European Association of Preventive Cardiology.” European Journal of Preventive Cardiology 28: 460–495. 33611446 10.1177/2047487320913379

[jan70023-bib-0002] Ang, D. T. Y. , and C. Berry . 2021. “What an Interventionalist Needs to Know About INOCA.” Interventional Cardiology: Reviews, Research, Resources 16: e32. 10.15420/icr.2021.16. PMC8674629 34950239

[jan70023-bib-0003] Asher, A. , R. Ghelani , G. Thornton , et al. 2019. “UK Perspective on the Changing Landscape of Non‐Invasive Cardiac Testing.” Open Heart 6: e001186. 31908814 10.1136/openhrt-2019-001186 PMC6927513

[jan70023-bib-0004] Beatty, A. L. , T. M. Beckie , J. Dodson , et al. 2023. “A New Era in Cardiac Rehabilitation Delivery: Research Gaps, Questions, Strategies, and Priorities.” Circulation 147: 254–266. 36649394 10.1161/CIRCULATIONAHA.122.061046 PMC9988237

[jan70023-bib-0005] Bove, K. B. , M. Nilsson , L. R. Pedersen , et al. 2020. “Comprehensive Treatment of Microvascular Angina in Overweight Women–A Randomized Controlled Pilot Trial.” PLoS One 15: e0240722. 33151955 10.1371/journal.pone.0240722 PMC7644075

[jan70023-bib-0006] Braun, V. , and V. Clarke . 2013. “Successful Qualitative Research: A Practical Guide for Beginners.”

[jan70023-bib-0007] British Association for Cardiovascular Prevention and Rehabilitation . 2023. “Standards and Core Components for Cardiovascular Disease Prevention and Rehabilitation.” https://www.bacpr.org/resources/publications.

[jan70023-bib-0008] Clark, A. M. , R. S. Barbour , M. White , and P. D. MacIntyre . 2004. “Promoting Participation in Cardiac Rehabilitation: Patient Choices and Experiences.” Journal of Advanced Nursing 47: 5–14. 15186462 10.1111/j.1365-2648.2004.03060.x

[jan70023-bib-0009] Cowie, A. , J. Buckley , P. Doherty , et al. 2019. “Standards and Core Components for Cardiovascular Disease Prevention and Rehabilitation.” Heart 105: 510–515. 30700518 10.1136/heartjnl-2018-314206 PMC6580752

[jan70023-bib-0010] Dalal, H. M. , R. S. Taylor , K. Jolly , et al. 2019. “The Effects and Costs of Home‐Based Rehabilitation for Heart Failure With Reduced Ejection Fraction: The REACH‐HF Multicentre Randomized Controlled Trial.” European Journal of Preventive Cardiology 26: 262–272. 30304644 10.1177/2047487318806358 PMC6376602

[jan70023-bib-0011] Dibben, G. , J. Faulkner , N. Oldridge , et al. 2021. “Exercise‐Based Cardiac Rehabilitation for Coronary Heart Disease.” Cochrane Database of Systematic Reviews 11: CD001800. 10.1002/14651858.CD001800.pub4. 34741536 PMC8571912

[jan70023-bib-0012] Ford, T. J. , S. Bethany , G. Richard , et al. 2018. “Stratified Medical Therapy Using Invasive Coronary Function Testing in Angina.” Journal of the American College of Cardiology 72: 2841–2855. 30266608 10.1016/j.jacc.2018.09.006

[jan70023-bib-0013] Gulati, M. , N. Khan , M. George , et al. 2023. “Ischemia With no Obstructive Coronary Artery Disease (INOCA): A Patient Self‐Report Quality of Life Survey From INOCA International.” International Journal of Cardiology 371: 28–39. 36162521 10.1016/j.ijcard.2022.09.047

[jan70023-bib-0014] Humphreys, H. , D. Paddock , S. Brown , et al. 2024. “Living With Myocardial Ischaemia and no Obstructive Coronary Arteries: A Qualitative Study.” Open Heart 11: e002569. 10.1136/openhrt-2023-002569. 38331473 PMC10860068

[jan70023-bib-0015] Hurdus, B. , T. Munyombwe , T. B. Dondo , et al. 2020. “Association of Cardiac Rehabilitation and Health‐Related Quality of Life Following Acute Myocardial Infarction.” Heart 106: 1726–1731. 32826289 10.1136/heartjnl-2020-316920 PMC7656151

[jan70023-bib-0016] Kissel, C. K. , and D. Nikoletou . 2018. “Cardiac Rehabilitation and Exercise Prescription in Symptomatic Patients With Non‐Obstructive Coronary Artery Disease—A Systematic Review.” Current Treatment Options in Cardiovascular Medicine 20: 1–12. 30121850 10.1007/s11936-018-0667-2 PMC6105244

[jan70023-bib-0017] Kunadian, V. , A. Chieffo , P. G. Camici , et al. 2020. “An EAPCI Expert Consensus Document on Ischaemia With Non‐Obstructive Coronary Arteries in Collaboration With European Society of Cardiology Working Group on Coronary Pathophysiology & Microcirculation Endorsed by Coronary Vasomotor Disorders International Study Group.” European Heart Journal 41: 3504–3520. 32626906 10.1093/eurheartj/ehaa503 PMC7577516

[jan70023-bib-0018] Maddox, T. M. , M. A. Stanislawski , G. K. Grunwald , et al. 2014. “Nonobstructive Coronary Artery Disease and Risk of Myocardial Infarction.” JAMA 312: 1754–1763. 25369489 10.1001/jama.2014.14681 PMC4893304

[jan70023-bib-0019] National Audit of Cardiac Rehabilitation . 2019. National Audit of Cardiac Rehabilitation (NACR) Quality and Outcomes Report 2019. British Heart Foundation.

[jan70023-bib-0020] NHS England . 2019. “The NHS Long Term Plan.”

[jan70023-bib-0021] Nordenskjöld, A. M. , T. Baron , K. M. Eggers , T. Jernberg , and B. Lindahl . 2018. “Predictors of Adverse Outcome in Patients With Myocardial Infarction With Non‐Obstructive Coronary Artery (MINOCA) Disease.” International Journal of Cardiology 261: 18–23. 29563017 10.1016/j.ijcard.2018.03.056

[jan70023-bib-0022] Powell, R. , G. McGregor , S. Ennis , P. K. Kimani , and M. Underwood . 2018. “Is Exercise‐Based Cardiac Rehabilitation Effective? A Systematic Review and Meta‐Analysis to Re‐Examine the Evidence.” BMJ Open 8: e019656. 10.1136/bmjopen-2017-019656 PMC5857699 29540415

[jan70023-bib-0023] Rahman, H. , D. Corcoran , M. Aetesam‐ur‐Rahman , S. P. Hoole , C. Berry , and D. Perera . 2019. “Diagnosis of Patients With Angina and Non‐Obstructive Coronary Disease in the Catheter Laboratory.” Heart 105: 1536–1542. 31366574 10.1136/heartjnl-2019-315042 PMC6774766

[jan70023-bib-0024] Taqueti, V. R. , S. D. Solomon , A. M. Shah , et al. 2018. “Coronary Microvascular Dysfunction and Future Risk of Heart Failure With Preserved Ejection Fraction.” European Heart Journal 39: 840–849. 29293969 10.1093/eurheartj/ehx721 PMC5939665

[jan70023-bib-0025] Taylor, R. S. , L. Long , I. R. Mordi , et al. 2019. “Exercise‐Based Rehabilitation for Heart Failure: Cochrane Systematic Review, Meta‐Analysis, and Trial Sequential Analysis.” JACC Heart Failure 7: 691–705. 31302050 10.1016/j.jchf.2019.04.023

[jan70023-bib-0026] Van Schalkwijk, D. L. , J. W. M. G. Widdershoven , S. Elias‐Smale , et al. 2023. “ShareHeart: A Patient Journey Map of Patients With Ischemia and Non‐Obstructive Coronary Artery Disease Based on Qualitative Research.” Journal of Clinical Nursing 32: 3434–3444. 35689371 10.1111/jocn.16409

[jan70023-bib-0027] Wheeler, A. , G. Schrader , G. Tucker , R. Adams , R. Tavella , and J. F. Beltrame . 2013. “Prevalence of Depression in Patients With Chest Pain and Non‐Obstructive Coronary Artery Disease.” American Journal of Cardiology 112: 656–659. 23711812 10.1016/j.amjcard.2013.04.042

