# Supplementary material for: Cardiovascular Prevention and Rehabilitation for Ischaemic Non‐Obstructive Coronary Artery Disease: Implementation Considerations From a Survey of UK Health Professionals
Source: J Adv Nurs. 2025 Jun 19;82(4):3716–25. doi: 10.1111/jan.70023 (PMC12994660; doi:10.1111/jan.70023)
Supplement: Supplementary file 2 — Appendix S2. [file JAN-82-3716-s001.docx]

**Appendix 2** - Survey

Thank you for taking the time to complete our brief survey. It should take approximately 15 minutes to complete. We have asked you to complete this survey because you are involved in the delivery of core (Phase III) cardiovascular prevention and rehabilitation, in the UK.

We are conducting a survey to help design a cardiovascular prevention and rehabilitation programme for people with non-obstructive coronary artery disease. This survey is open to people who work in a **UK** cardiovascular prevention and rehabilitation service because we want our findings to be applicable to a UK healthcare setting. The survey will help us to understand whether you think your cardiovascular prevention and rehabilitation programme has the resources to treat people with non-obstructive coronary artery disease. The survey also asks questions about whether you think your team currently have the skills to treat people with non-obstructive coronary artery disease, and what benefits you think people with non-obstructive coronary artery disease would get from participating in a cardiovascular prevention and rehabilitation programme. **You do not need to know anything about non-obstructive coronary artery disease to complete this survey**. **Answering ‘not sure’ to a question is as helpful as providing any other response.** Any information you provide will be helpful.

Only information that is essential to answer our research question will be collected and will be processed in accordance with the General Data Protection Regulation (2018). If you would like to withdraw from the study, just exit the web page. **We will keep the responses you have provide even if you don’t complete the whole survey.** On the next page, before you complete the survey, you will be asked to complete an informed consent form to confirm you are happy to participate in the study. If you would like any information about data protection or the study, please contact:

1. I have had the opportunity to read the study information on the previous page and, where appropriate, asked any questions and I am satisfied with the answers I had.
2. I understand that my participation is voluntary and that I am free to withdraw at any time without giving any reason.
3. I understand that I can end the survey at any time, but that any answers I have provided will be saved.
4. I understand that my personal information will be collected, stored and used by authorised individuals from Sheffield Hallam University.
5. I agree to take part in the above study.

Date questionnaire was completed __/__/__ (Automated)

**Screening questions**

Have you previously completed this questionnaire?

Yes / No

Do you work in in the UK?

Yes / No

Do you work in Core (Phase III) cardiovascular prevention and rehabilitation?

Yes / No

*-----------------------------------------If yes/no to any of the above, survey ends as appropriate------------------*

**Background Information**

1. Which country do you work in? (Please tick one)

England

Northern Ireland

Scotland

Wales

1. What is your professional background? (Please tick one)

Dietitian

Doctor (Physician)

Exercise Instructor

Exercise Physiologist

Nurse

Occupational Therapist

Physiotherapist

Psychologist

Other (Please Specify) __________

1. Which of the following roles best describes your position within your cardiovascular prevention and rehabilitation programme? (Please tick one)

Manager/Service Manager

Team leader

Staff member in Cardiovascular Rehabilitation

Assistant

Other__________

1. How long have you worked in cardiovascular prevention and rehabilitation?

______Years______Months

1. What is your highest level of academic qualification? (Please tick one)

PhD

MSc/MA

PGCert

BSc

Diploma

HNC

A Level

BTEC

HND

Other_______

1. What formal cardiovascular prevention and rehabilitation course have you completed? (tick all that apply)

BACPR/Other Specialist (Level 4) Exercise Instructor Qualification

Physical Activity and Exercise Short Course

Health Behaviour Change and Psychological Support in Cardiovascular Disease

Dietary Approaches in the Management of Cardiovascular Disease

Medical Risk Factor Management and Cardioprotective Therapies

Other______

**Section - Do UK cardiovascular prevention and rehabilitation programmes have capacity to provide treatment to patients with non-obstructive coronary artery disease?**

1. Does your programme currently accept referrals for patients with a primary diagnosis of one of the following conditions: (tick all that apply)

Arrhythmias

Angina

Coronary artery bypass graft

Elective percutaneous coronary intervention

Heart failure

Heart Transplant

Implantable Cardioverter Defibrillator

Left Ventricular Assist Device

Myocardial Infarction

Pacemaker

Peripheral Vascular Disease

Stroke

Transcatheter aortic valve implantation

Valve replacement

Other (Please Specify) _________

1. What components of cardiovascular prevention and rehabilitation do you typically offer to your patients? (tick all that apply)

Counselling

Dietetics or nutritional support

Exercise training

Health behaviour change support

Medication titration

Physical activity advice

Psychological support

Smoking cessation

Weight management services

Other (Please Specify) _________

1. Does your programme currently accept referrals for patients with a primary diagnosis of non-obstructive coronary artery disease? (please tick one answer that best describes this)

Non-obstructive coronary artery disease means people with endothelial dysfunction, microvascular remodelling, microvascular and epicardial spasm, vasomotor abnormalities and enhanced cardiac pain perception.

No

Yes – Rarely

Yes – Our service receives several referrals per year

Yes – Our service receives several referrals per month

Yes – At least weekly

*------------------------------------------------If ‘no’, redirect to question 11----------------------------------------------*

1. If your programme does accept referrals for people with non-obstructive coronary artery disease, is there a formal referral pathway or are any referrals you receive ‘ad-hoc’ or occasional? (please tick one)

Formal referral pathway

Ad-hoc referrals/occasional

1. If there were a formal referral process for patients with non-obstructive coronary artery disease, would you have capacity to assess them? (please tick one)

Yes – There would be no problems with capacity

Yes – But we would only have capacity for a limited number of patients

No – We would not have any capacity to see them

Not sure

Please briefly explain your answer_______________________________

1. If there was a formal referral process for patients with non-obstructive coronary artery disease, do you think that your service would have capacity to provide comprehensive cardiovascular prevention and rehabilitation to them? (Please tick one).

Comprehensive cardiovascular prevention and rehabilitation means all components recommended by the British Association for Cardiovascular Prevention and Rehabilitation standards, not just a single component, such as exercise.

Yes – There would be no problems with capacity

Yes – But we would only have capacity for a limited number of patients

No – We would not have any capacity to see them

Not sure

Please briefly explain your answer_______________________________

1. Would the cardiovascular prevention and rehabilitation programme need to be:

Centre-based or home-based (including technology-based)

Centre-based only

Home-based only (including technology-based)

Not sure

Please briefly explain your answer_______________________

1. What additional resources, if any, would you need to be able to provide comprehensive cardiovascular prevention and rehabilitation to people with non-obstructive coronary artery disease? (tick all that apply)

None

Additional venues/space

Equipment

Staff/Human Resources

Funding

Other (Please Specify) _________

**Section – What level of care should be provided to patients with non-obstructive coronary artery disease?**

In the ‘real world’, we understand that cardiovascular prevention and rehabilitation interventions can be personalised and that not all patients receive the same treatment. However, to help us design a research trial, where all participants get the same intervention, we would like to know what level of care you think should be offered to people with non-obstructive coronary artery disease.

1. Do you think people with non-obstructive coronary artery disease would benefit from participating in cardiovascular prevention and rehabilitation?

Yes

No

Not Sure

1. In a research trial, what components of cardiovascular prevention and rehabilitation do you think should be offered to people with non-obstructive coronary artery disease? (tick all that apply)

Not Sure

Counselling

Dietetics or nutritional support

Exercise training

Health behaviour change support

Medication titration

Physical activity advice

Psychological support

Smoking cessation

Weight management services

Other (Please Specify) _________

Please briefly explain your answer_______________________________

1. In total, how many weeks of cardiovascular prevention and rehabilitation treatment do you think people with non-obstructive coronary artery disease should be offered in a research trial?

Not Sure

______ weeks

1. In a research trial, how many sessions of supervised cardiovascular prevention and rehabilitation do you think people with non-obstructive coronary artery disease should receive *per week*?

Not Sure

______ sessions

1. In a research trial, how many sessions of un-supervised cardiovascular prevention and rehabilitation do you think people with non-obstructive coronary artery disease should receive *per week*?

Not Sure

______ sessions

1. In a research trial, should people with non-obstructive coronary artery disease be offered (tick all that apply):

Hybrid of centre-based and home-based cardiovascular prevention and rehabilitation

Centre-based cardiovascular prevention and rehabilitation

Home-based cardiovascular prevention and rehabilitation
Online cardiovascular prevention and rehabilitation

Not sure

If possible, please briefly explain your answer_______________________________

**Section - Knowledge possessed by cardiovascular prevention and rehabilitation healthcare professionals about treating patients with INOCA.**

1. Have you ever, in any job, provided cardiovascular prevention and rehabilitation programmes to someone with non-obstructive coronary artery disease? This does not necessarily have to have been their primary reason for attending cardiovascular prevention and rehabilitation.

Yes /No

*------------------------------------------------If ‘no’, redirect to question 24----------------------------------------------*

1. If you have ever provided cardiovascular prevention and rehabilitation programme to someone with non-obstructive coronary artery disease, were you confident managing their condition?

Yes – Completely confident

Yes – But I would have liked additional training

No – I wasn’t always able to provide a good level of support to the patient

1. On a scale of 0 to 10, with 0 meaning not confident at all, and 10 being completely confident, how confident were you when managing patients with non-obstructive coronary artery disease?

0 1 2 3 4 5 6 7 8 9 10

1. Do you think that you currently know enough about non-obstructive coronary artery disease to provide an effective cardiovascular prevention and rehabilitation programme to patients with this condition?

Yes

No

Not Sure

1. Do you think that that the majority of your colleagues know enough about non-obstructive coronary artery disease to provide an effective cardiovascular prevention and rehabilitation programme to patients with this condition?

Yes

No

Not Sure

1. If you do not feel you have enough knowledge about non-obstructive coronary artery disease, what would you consider doing to enhance your knowledge?

Speak to colleagues to find out more

Consult internet for information

Search for and read healthcare journals on this topic

Attend educational CPD session

Sign up for a module at university to learn more

Other (please specify) ________________

**Section - In principle, will any of the cardiovascular prevention and rehabilitation programmes participate in a future definitive multi-centre RCT?**

1. If there was a funded research project that investigated the benefits of cardiovascular prevention and rehabilitation, in principle, would you be willing to take part? This would mean providing treatment to patients.

Yes

No

Not sure

Are there any specific recommendations you would want our research team to consider when designing and implementing a research project that assesses the benefits of cardiovascular prevention and rehabilitation for people with non-obstructive coronary artery disease?

No

Yes - Please briefly describe__________________________________________________________

*Survey ends*

*Thank you for taking the time to complete our survey. Once published, we plan to share our findings through the British Association for Cardiovascular Prevention and Rehabilitation mailing list. Final publication of our findings may take more than a year from when this survey opened.*

**Appendix 3** – Examples Promotional Material

**E-mail**

| \| \| Dear XXXX  **INOCA Survey**    Ischaemic non-obstructive coronary artery disease (INOCA) is a term describing a group of conditions including endothelial dysfunction, microvascular remodelling, microvascular and epicardial spasm and vasomotor abnormalities. People with INOCA might benefit from cardiovascular prevention and rehabilitation. The BACPR are supporting research, funded by the BHF CRC, which aims to develop a research trial that will investigate the benefits of cardiovascular prevention and rehabilitation for people with INOCA.   One part of the research programme is a survey. The findings of the survey will help design a cardiovascular prevention and rehabilitation programme for people with INOCA. The survey is only open to people who work in a UK cardiovascular prevention and rehabilitation service because we want our findings to be applicable to a UK healthcare setting. You do not need to know anything about INOCA to complete this survey. Answering ‘not sure’ to a question is as helpful as providing any other response. Any information you provide will be helpful.   The survey can be completed on a desktop/laptop or your smart phone and should take around 10 minutes to complete. More than one person per team can respond to the survey. **Once the findings of the survey have been collated and analysed, we will ensure that BACPR members are told what the findings are in a future e-mail.** This could take approximately 18 months. \| \| --- \| \| \| --- \| --- \| |
| --- | --- | --- |

| \| [**Click here to complete survey**](https://eur01.safelinks.protection.outlook.com/?url=https%3A%2F%2Fbacpr.us17.list-manage.com%2Ftrack%2Fclick%3Fu%3De10a095d4eeaac45b66c9cee7%26id%3Db3d0608bc1%26e%3D3f845844f1&data=05%7C01%7Cs.nichols%40rgu.ac.uk%7C2bec5b54160545f2c73808dbe4505593%7C51a0a69c0e4f4b3db64212e013198635%7C0%7C0%7C638354805908746228%7CUnknown%7CTWFpbGZsb3d8eyJWIjoiMC4wLjAwMDAiLCJQIjoiV2luMzIiLCJBTiI6Ik1haWwiLCJXVCI6Mn0%3D%7C3000%7C%7C%7C&sdata=5fCvJ4DRdeRBzJSmJo4NINcG6CzP2vTFqjWPJs5M2ac%3D&reserved=0) \| \| --- \| |
| --- | --- |

| \| \| Thank you for taking the time to consider taking part in this research. Your support is appreciated.   Kind Regards \| \| --- \| \| \| --- \| --- \| |
| --- | --- | --- |

**Twitter**

1. **We are developing a project to test #cardiacrehab in non-obstructive coronary artery disease. We'd like to know if your UK PIII programme has the resources to deliver this in future practice 👉https://shusls.eu.qualtrics.com/jfe/form/SV_0T9QNgkliBESLd4…**
2. **We've had a great response to our survey asking if UK PIII programmes could deliver #cardiacrehab to people with non-obstructive coronary artery disease in future practice. There's still time to take part! https://shusls.eu.qualtrics.com/jfe/form/SV_0T9QNgkliBESLd4**

**Appendix 4** – Respondents job role (Table 1), educational qualifications (Table 2), and cardiovascular prevention and rehabilitation courses completed (Table 3)

**Table 1 -** Job role of respondents

| **Job Role** |  | n=108 |
| --- | --- | --- |
| Manager/Service Manager | | 16 (14.8) |
| Team Leader |  | 31 (28.7) |
| Staff Member | | 59 (54.6) |
| Assistant |  | 1 (0.9) |
| Honorary Medical Director | | 1 (0.9) |

**Table 2 -** Highest level of academic

| **Qualification** | | n=108 |
| --- | --- | --- |
| PhD |  | 1 (0.9) |
| Medical Doctorate | | 1 (0.9) |
| MSc/MA |  | 28 (25.9) |
| Post-Graduate Diploma | | 2 (1.9) |
| Post-Graduate Certificate | | 5 (4.6) |
| BSc |  | 54 (50.0) |
| Diploma |  | 9 (8.3) |
| A Level |  | 1 (0.9) |
| Cert Ed |  | 1 (0.9) |
| Registered Nurse Training | | 3 (2.8) |
| None |  | 1 (0.9) |
| Unclear response | | 2 (0.9) |

**Table 3 -** Cardiovascular prevention and rehabilitation courses completed

| **Courses completed** | | | | | |  | n=108 |
| --- | --- | --- | --- | --- | --- | --- | --- |
| Level 4 Exercise Instructor Course | | |  |  |  |  | 29 (26.9) |
| BACPR Physical Activity Short Courses (Part A and B) | | | |  |  |  | 70 (64.8) |
| BACPR Health Behaviour Change and Psychological Support in Cardiovascular Disease | | | | | | | 48 (44.4) |
| BACPR Dietary Approaches in the Management of Cardiovascular Disease | | | | | |  | 29 (26.9) |
| BACPR Medical Risk Factor Management | | | |  |  |  | 35 (32.4) |
| BACPR Physical Activity and Exercise in Heart Failure Course | | | | |  |  | 2 (1.9) |
| BACPR Assessing Functional Capacity | | |  |  |  |  | 4 (3.7) |
| BACPR Physical Activity and Exercise in Type 2 Diabetes | | | | |  |  | 2 (1.9) |
| Clinical Examination and Diagnosis (Level 7) | | | |  |  |  | 1 (0.9) |
| Heart Failure Course | |  |  |  |  |  | 2 (1.9) |
| Heart Manual Training | |  |  |  |  |  | 1 (0.9) |
| Angioplasty Plan Training | |  |  |  |  |  | 1 (0.9) |
| BACPR Courses | |  |  |  |  |  | 3 (2.8) |
| Unspecified University modules | | |  |  |  |  | 4 (3.7) |
| Undergraduate Module | |  |  |  |  |  | 1 (0.9) |
| Unclear response | |  |  |  |  |  | 1 (0.9) |
| None |  |  |  |  |  |  | 12 (11.1) |

**Appendix 5** – Higher Order themes with illustrative quotes

| Higher Order Theme | Subtheme | Description | Illustrative Quote(s) |
| --- | --- | --- | --- |
| Resources | Sufficient resources for assessment and CPR | Some respondents reported having adequate staff or anticipated low demand, enabling them to assess and treat patients with INOCA. | “I would assume numbers would be low, and we could therefor absorb them into our caseload”  “We are already seeing this group of patients”  “Our contract allows all referrals to be included”  “Numbers wouldn’t be huge” |
| Resources | Insufficient resources | Many reported staffing shortages, limited clinic space, long waiting lists, or residual impacts from COVID-19 as barriers to assessment and CPR provision. | “We don’t offer comprehensive rehab to our current cohort so would need more staff e.g. psychology, dietetics, and access to space for clinics and exercise”  “35hrs per week shared by 2 physios, no other capacity at present to see more patients”  “Understaffed (no physio or exercise professional), space for exercise classes limited”  “Several vacancies at present”  “Small team”  Because of the pandemic, we are struggling to offer all the normal components of rehabilitation” |
| Resources | Uncertainty or unclear demand | Uncertainty around local referral pathways or patient numbers made it difficult for services to plan provision. | “There is no local data on the number of patients with INOCA who would be eligible for CR”  “This would need to be a board wide decision based on projected numbers”  “It would depend on the number of referrals” |
| Programme Format | Preference-based flexibility | Respondents supported providing centre-based, home-based, or hybrid models depending on patient need or choice. | “They should have the choice of all options, but anecdotal experience suggests our patients benefit most from a combination of group and home based”  “This is decided on individual basis – can’t make blanket decisions”  “Patient choice so it allows their programme to be individualised to meet their goals” |
| Programme Format | Determined by current provision | In some services, programme formats were constrained to either face-to-face or home-based, based on existing models or available space. | “Home-based is only available at the moment as gym space not available”  “We run sessions throughout the community at leisure centres and clinics”  “Would be able to manage home based/tech options better with limited capacity” |
